# Supplementary material for: Perifocal Zone of Brain Gliomas: Application of Diffusion Kurtosis and Perfusion MRI Values for Tumor Invasion Border Determination
Source: Cancers (Basel). 2023 May 15;15(10):2760. doi: 10.3390/cancers15102760 (PMC10216555; doi:10.3390/cancers15102760)
Supplement: Supplementary file 1 [file cancers-15-02760-s001.zip › cancers-2262653-supplementary.pdf]

Supplementary material

Supplemental table S1. Mean ASL and DKI parameter values in the combined patient group.

	ROI1		ROI2		ROI3		ROI4	
	mean	sd	mean	sd	mean	sd	mean	sd
CBF	87,68	51,69	28,50	19,15	28,83	19,94	21,22	7,82
AK	0,44	0,11	0,35	0,09	0,34	0,18	0,38	0,10
AWF	0,24	0,06	0,23	0,07	0,43	0,11	0,58	0,09
AxEAD	1,87	0,35	2,14	0,43	1,80	0,29	1,92	0,17
AxIAD	0,47	0,19	0,66	0,18	0,82	0,23	1,02	0,18
FA	0,15	0,07	0,17	0,07	0,32	0,10	0,44	0,09
KA	0,18	0,12	0,20	0,07	0,36	0,11	0,54	0,13
MD	1,37	0,34	1,57	0,40	1,03	0,21	0,92	0,07
MK	0,62	0,16	0,54	0,14	0,94	0,26	1,40	0,23
RadEAD	1,61	0,35	1,79	0,40	1,36	0,21	1,41	0,11
RadIAD	0,11	0,05	0,17	0,06	0,19	0,06	0,18	0,05
RK	0,75	0,31	0,70	0,24	1,57	0,64	2,80	0,78
TORT	1,18	0,10	1,21	0,10	1,33	0,14	1,38	0,16

Supplemental table S2. Mean ASL and DKI parameter values in the glioblastoma group.

	ROI1		ROI2		ROI3		ROI4	
	mean	sd	mean	sd	mean	sd	mean	sd
CBF	100,74	53,08	29,99	21,77	28,62	19,41	20,68	7,44
AK	0,45	0,11	0,35	0,09	0,33	0,20	0,39	0,10
AWF	0,25	0,06	0,24	0,07	0,43	0,12	0,57	0,09
AxEAD	1,79	0,31	2,06	0,42	1,78	0,30	1,90	0,17
AxIAD	0,48	0,20	0,68	0,18	0,80	0,23	1,00	0,18
FA	0,16	0,07	0,18	0,07	0,32	0,10	0,42	0,09
KA	0,19	0,14	0,21	0,08	0,35	0,11	0,52	0,11
MD	1,28	0,28	1,49	0,38	1,01	0,23	0,94	0,08
MK	0,65	0,16	0,57	0,14	0,96	0,27	1,38	0,23
RadEAD	1,52	0,29	1,71	0,38	1,36	0,23	1,42	0,11
RadIAD	0,12	0,06	0,17	0,06	0,19	0,07	0,18	0,05
RK	0,79	0,34	0,74	0,24	1,60	0,67	2,70	0,75
TORT	1,19	0,11	1,22	0,10	1,31	0,14	1,35	0,07

Supplemental table S3. Differences of ASL and DKI parameter values between ROIs in the combined patient group.

p-values for the combined patient group						
	1vs2		2vs3		3vs4	
CBF	**2,43E-11**	**<0,001**	0,945834		0,108193	
RadIAD	**1,13E-05**	**<0,001**	0,141609		0,397736	
AxIAD	**7,42E-05**	**<0,001**	**0,00087**	**<0,001**	**0,000177**	**<0,001**
AK	**0,000179**	**<0,001**	0,734316		0,473958	
AxEAD	0,004082		**0,000302**	**<0,001**	0,033595	
MK	0,011534		**7,36E-13**	**<0,001**	**9,11E-13**	**<0,001**
KA	0,017497		**3,15E-09**	**<0,001**	**2,24E-09**	**<0,001**
MD	0,021982		**7,00E-11**	**<0,001**	**0,00013**	**<0,001**
RadEAD	0,037704		**9,98E-08**	**<0,001**	0,064269	
TORT	0,05494		**8,13E-08**	**<0,001**	0,282187	
FA	0,102018		**3,95E-11**	**<0,001**	**1,25E-06**	**<0,001**
RK	0,131204		**9,24E-13**	**<0,001**	**1,23E-11**	**<0,001**
AWF	0,376529		**2,57E-13**	**<0,001**	**1,39E-09**	**<0,001**

Supplemental table S4. Differences of ASL and DKI parameter values between ROIs in the glioblastoma group.

p-values for the glioblastoma group						
	1vs2		2vs3		3vs4	
CBF	**6,30E-10**	**<0,001**	0,898236		0,074692	
RadIAD	**0,000265**	**<0,001**	0,493102		0,719149	
AxIAD	**0,000248**	**<0,001**	0,023567		**0,00082**	**<0,001**
AK	**0,000324**	**<0,001**	0,689994		0,774744	
AxEAD	0,006481		0,011868		0,059062	
MK	0,022376		**6,18E-09**	**<0,001**	**1,37E-08**	**<0,001**
MD	0,028703		**1,04E-07**	**<0,001**	0,019102	
KA	0,042597		**2,07E-06**	**<0,001**	**1,90E-07**	**<0,001**
RadEAD	0,044252		**5,54E-05**	**<0,001**	0,027381	
TORT	0,124295		**2,81E-05**	**<0,001**	0,440431	
FA	0,172436		**8,36E-08**	**<0,001**	**7,22E-05**	**<0,001**
RK	0,239239		**1,31E-08**	**<0,001**	**9,16E-08**	**<0,001**
AWF	0,421577		**4,78E-09**	**<0,001**	**4,11E-06**	**<0,001**
